# Candida auris in the Age of Resistance

**DOI:** 10.7759/cureus.10334

**Published:** 2020-09-09

**Authors:** Michael Oh, Jonathan Heyl, Benson A Babu

**Affiliations:** 1 Medicine, Lake Erie College of Osteopathic Medicine, Auburndale, USA; 2 Medicine, Lake Erie College of Osteopathic Medicine, Bradenton, USA; 3 Hospital Medicine, Northwell Health, New York, USA

**Keywords:** candida auris

## Abstract

*Candida auris *(*C. auris)* is an opportunistic ascomycetous budding yeast that has been emerging as an invasive, multidrug-resistant pathogen over the past 10 years since its discovery. This fungi is the first to be labeled as a public health threat according to the Centers for Disease Control (CDC) and has since become a major problem in the United States. This serves as a detailed overview of the various factors contributing to the pathogenicity of *C. auris*.

## Introduction and background

*Candida auris* has come into the spotlight as a dominant, drug-resistant, invasive organism over the past 10 years. It is the first fungal organism labeled as a public health threat, and its troublesome nature can be attributed to unreliable diagnostic methods, complexities of its pathogenicity, and lack of a gold standard treatment and hospital management. These factors contribute to a high mortality rate and rate of antifungal resistance. Identifying the current and initial epidemiological spread is crucial for discovering potential treatment options.

## Review

Epidemiology and re-emergence

The first discovery of *Candida auris (C. Auris)* was in 2009 in Japan from the external ear canal of an inpatient, although a retrospective review of isolates points to South Korea having the first known infection, dating back to 1996. Identification was done with ribosomal ribonucleic acid (rRNA) sequencing and biochemical analyses showing a new Candida species. From 2009 to 2011, two hospitals in Delhi, India, were also found to contain patients with candidemia with high mortality rates [1]. The global spread was imminent, as cases began to break out in 2016, first in Southeast Asia and then in London. It was not until 2017 that the United States publicly renounced *C. auris* as a justifiable threat as a multi-drug resistant organism. There are now cases recorded in every continent except for Antarctica [2].

As of October 31, 2019, the total number of cases of *C. Auris* recorded by the Centers for Disease Control (CDC) in the United States totaled 911, which is an eerie rise from the 77 reported cases reported as of May 2017 [3]. The epicenter of the spread of this deadly organism seems to take its roots in the states of New York and New Jersey, along with Chicago, which remain the source of nearly all the confirmed cases [4].

A common underlying theme is that the patients that contracted *C. Auris* had high exposures to healthcare facilities along with comorbid conditions. Although the method of transmission within and between healthcare facilities has not been confirmed, it seems plausible that spread by skin or mucosal colonization is a factor, as the organism has been found in areas such as sinks, furniture, and medical equipment [5]. In fact, the CDC has recommended precautions similar to the handling of other transmissible diseases such as those caused by *Clostridium difficile*: the housing of infected patients in private rooms, good hand hygiene, and so on. Despite these precautions, the number of infections remains to be on the rise. This makes it of the utmost importance to swiftly identify infections with even prompter treatment [6].

Classification of Candida auris

Candida auris is an opportunistic, ascomycetous budding yeast that was first discovered in 2009 in Japan [7]. This discovery could take place because it was able to grow in the presence of an echinocandin. It is commonly confused with similar-looking organisms such as *Candida haemulonii*, *Candida duobushaemulonii,* and *Rhodotorula glutinis*. *Candida auris *typically grows best on CHROMagar^© ^(CHROMagar Microbiology, France) at 40-42℃ and can appear white, red, pink, or purple, which differentiates it from other members of the genus Candida. However, to correctly identify *Candida auris*, researchers used a deoxyribonucleic (DNA) sequence analysis to differentiate this new strain of fungi [7]. 

Most subtypes of Candida are identified using Vitek 2 YST^© ^(bioMérieux, Marcy-l'Étoile, France), API 20C^© ^(bioMérieux), API ID 32 C^© ^(bioMérieux), BD Phoenix yeast identification system^© ^(Becton, Dickinson and Company Franklin Lakes, New Jersey), MicroScan^© ^(Beckman Coulter, Pasadena, California), or RapID Yeast Plus^© ^(Innovative Diagnostic Systems, Saint-Eustache, Quebec, Canada) according to the CDC. *C. auris* is typically initially identified using VITEK 2^©^. This identification method commonly causes misidentification since *C. auris* appears so similarly to *Candida lusitaniae *and Candida famata at the early stages. It is recommended by the CDC that follow-up cultures on cornmeal agar be done when *Candida auris* is suspected. If the culture grows without hyphae or pseudohyphae, the culture is most likely *Candida auris*, as *Candida lusitaniae* and *Candida famata* have these adaptations. Misidentification can still be an issue at this point as well because some strains of *C. auris* have recently been found to have hyphae and pseudohyphae. 

*Mechanism of Pathogenicity*

The first three infectious causes of *C. auris *in South Korea lead to fungemia, resulting in death in two of these patients [1]. Multiple factors, including difficulty to identify *C. auris*, antibiotic resistance, and a plethora of virulence factors, make the mortality rate particularly high in infected individuals [8]. One of the factors that make *C. auris* so deadly is that it can evade the response of the innate immune system by disabling neutrophil extracellular traps (NETs) in comparison to *C. albicans* [9]. *C. auris* has multiple other virulence factors, including lipases, mannosyl transferases, oligopeptide transporters, and the ability to develop a biofilm [10]. Lipases are a catalyst for the hydrolysis of ester bonds of triacylglycerols, which allows fatty acids to be released [11]. Candida species use these to destroy epidermal and epithelial tissues [12]. The glycosyltransferase, mannosyl transferase, is also very important, as it aids in the adhesion of *C. auris* to the epithelium of its host and other clusters of *C. auris* [13].

Known virulence factors and difficulty with the identification of *C. auris* have made this fungus incredibly dangerous when it infects a host, but arguably the most concerning part of *C. auris* is its high resistance profile to popular antifungals. Over 90% of known strains of *C. auris *are resistant to fluconazole while up to 73% of strains are resistant to voriconazole [11]. Another study found that select isolates of C. auris were resistant to the common last resort drug amphotericin B at a rate up to 35% [12]. Fortunately, *C. auris* is still susceptible to multiple other triazoles and the majority of echinocandins [12]. The primary reason that explains the wide range of resistance is due to the overexpression of adenosine 5'-triphosphate (ATP)-binding cassette transporter (ABC) genes that were discovered in the *C. auris* genome [10]. ABC transporter genes use ATP binding and hydrolysis to provide energy for the translocation of substrates, which leads to increased expulsion of the drug out of its cells.

Treatment

There are a number of steps to follow when facing a case of *Candida auris*. First, it is important to have an immediate consultation with an infectious disease specialist in the facility. Measures to control infection should be in place once a case is identified while treating it and after treatment.

In adults, the current guideline for initial therapy is with an echinocandin drug as an anidulafungin loading dose of 200 mg intravenous (IV) followed by 100 mg IV daily, caspofungin loading dose 70 mg/m^2^/day IV, then 50 mg/m^2^ IV daily or micafungin 100 mg IV daily (2 mg/kg/day IV).

Given *C. auris*’s fast development of resistance, once a patient is identified with the infection and started on treatment, follow-up cultures and repeat susceptibility testing should be conducted. The majority of cases in the United States have been susceptible to echinocandins, however, there are cases of resistance reported. In those cases, amphotericin B (5 mg/kg daily) could be considered if the patient is clinically unresponsive to echinocandin treatment or has persistent fungemia for more than five days [14].

In affected neonates and infants younger than two months, the initial treatment is amphotericin B deoxycholate 1 mg/kg daily; if unresponsive, then liposomal amphotericin B 5 mg/kg could be considered. Cases, where the central nervous system is not compromised, may consider echinocandins such as caspofungin 25 mg/m^2^/day IV or micafungin 10 mg/kg/day IV.

The CDC does not recommend the treatment of *C. auris* identified from noninvasive sites (such as respiratory tract, urine, and skin colonization) when there is no evidence of infection. Similar to recommendations for other Candida species, treatment is generally only indicated if the clinical disease is present. However, infection control measures should be used for all patients with *C. auris*, regardless of the source of the specimen [15].

Management and Prevention

When facing a suspected or confirmed case of *C. auris*, a report to the CDC is warranted [16]. The patient should be placed in a single room under contact precautions with reinforced and enhanced hand hygiene practices with the recommended products, which include alcohol-based hand sanitizer, or if the hands are visibly soiled with soap and water [17]. Thorough environmental cleaning and disinfection of the patient care area should continue in order to prevent further spread of the organism. Contact tracing and testing of other potential colonized patients with *C. auris* is recommended through a swab of the axilla and groin to assess skin colonization, with an emphasis on roommates and those with the longest overlapping contact with the case-patient. Microbiology records should be reviewed (at least for the preceding one year) for suspected or confirmed cases of *C. auris *at the institution. Although the duration of infection is not necessarily known, it is recommended that patients be reassessed periodically every few months, which includes testing the axilla and groin sites along with sites yielding *C. auris *on previous cultures. It would be deemed safe to discontinue *C. auris* infection control precautions if a patient’s repeat swab is negative a week following an initial negative test [17].

Communication between facilities about infection status and screening protocol for newly identified patients is important. Environmental disinfection serves to be very important as well because *C. auris* persists on various surfaces in the healthcare environment, including bedside tables, bed rails, and window sills, and on mobile surfaces that travel in between rooms such as glucometers, blood pressure cuffs, ultrasound machines, crash carts, and nursing carts [17]. A disinfecting agent has not been standardized yet, but recent evidence has shown that quaternary ammonium compounds routinely used for disinfecting may not be effective against *C. auris*. Until we receive further information regarding the topic, the CDC recommends the use of an Environmental Protection Agency (EPA)-registered disinfectant with proven efficacy against *Clostridium difficile* spores.

## Conclusions

*Candida auris *is a highly infectious opportunistic yeast that has been labeled as a major public threat by the CDC, which is attributed to its highly infectious nature, antibiotic resistance, and identification difficulties. As it is resistant to most of the fluconazole classes, the mainstay of treatment includes using an echinocandin such as micafungin. If the infection has not been cleared in five days or is recurring, amphotericin B should be considered. Patients should be placed in single rooms with specific contact precautions to avoid spread. Notification to public health agencies should also be done as soon as possible. It is evident that treatment should be implemented early, with serial follow-up and investigation of colonization throughout a patient's treatment course.
